# Investigating the Influential Factors of Mild Water-Filtered Infrared-A Whole-Body Hyperthermia for Pain Relief in Fibromyalgia: A Mixed-Methods Approach Focusing on Predictors and Patient Perspectives

**DOI:** 10.3390/biomedicines11112949

**Published:** 2023-11-01

**Authors:** Özlem Öznur, Christoph Schlee, Sandra Utz, Jost Langhorst

**Affiliations:** 1 Department of Internal and Integrative Medicine, Sozialstiftung Bamberg, 96049 Bamberg, Germany; oezlem.oeznur@sozialstiftung-bamberg.de (Ö.Ö.); christoph.schlee@sozialstiftung-bamberg.de (C.S.); sandra.utz@sozialstiftung-bamberg.de (S.U.); 2 Department of Integrative Medicine, Medicinal Faculty, University of Duisburg-Essen, 96049 Bamberg, Germany; 3 Department of Sociology, University of Bamberg, 96052 Bamberg, Germany

**Keywords:** fibromyalgia, whole-body hyperthermia, randomized controlled trial, integrative medicine, mixed methods

## Abstract

Fibromyalgia syndrome (FMS) is a chronic condition characterized by chronic widespread pain, persistent fatigue, and disrupted sleep, significantly impacting well-being. Mild water-filtered infrared-A (wIRA) whole-body hyperthermia (WBH) is emerging as a promising pain management approach to FMS. Within the present randomized controlled trial (ClinicalTrials ID: NCT05135936), FMS patients underwent six sessions of mild wIRA-WBH over 3 weeks. Their pain levels were assessed at baseline and at week 12, while body core temperature and plateau phase duration were monitored during WBH. Qualitative interviews were conducted at week 12. Results from this mixed-methods study revealed that baseline pain intensity and plateau phase duration significantly predicted pain intensity at week 12. Thematic analysis of the interviews revealed diverse patient experiences with the treatment, with all patients reporting improvements in perceived pain and overall well-being. The onset and duration of pain relief varied among individuals. Overall, the findings suggest that the duration of the plateau phase may serve as an indicator for long-term pain reduction, although individual factors may influence treatment outcomes. Despite varying experiences, a prevailing trend of positive patient evaluations emerged. This study sheds light on the potential of wIRA-WBH as a therapeutic option for alleviating pain for and enhancing the well-being of FMS patients.

## 1. Introduction

Fibromyalgia syndrome (FMS) is a chronic condition whose main symptoms are chronic widespread pain, physical and/or mental fatigue, and non-restorative sleep [1,2]. The worldwide prevalence varies between 0.2 and 6.6% [3] and affects mostly women, especially in older/middle age [4]. FMS can be classified as a functional somatic syndrome [5]. It is often accompanied by diverse vegetative and functional symptoms, such as joint pain with morning stiffness, headaches, anxiety, depressiveness, or lower abdominal pain or cramps.

There are certain biological factors associated with FMS. These include inflammatory rheumatic diseases, vitamin D deficiency, and gene polymorphisms of the 5HT2 receptor. Some adults can carry fibromyalgia from childhood on [6,7,8]. Obesity, smoking, physical inactivity, childhood physical/sexual abuse, sexual violence in adulthood, and depressive disorders are psychosocial factors that accompany FMS [9].

Still unclear, however, are the factors for the underlying pathophysiology. Diverse factors are discussed: altered central pain processing (central sensitization) [10], alterations in the central nervous neurotransmitters [11], dysfunction of the sympathetic [12] or parasympathetic nervous system [13], small fiber pathology [9,14], and abnormality of microcirculation [15] are considered possible causes. The enhanced pain sensitivity and persistence of widespread pain in people with fibromyalgia may be caused by changes in the central processing of sensory input and deficiencies in endogenous pain inhibition [16].

The treatment of FMS is symptomatic and often perceived as insufficient due to the lack of long-term effective interventions and due to prescription of medication (esp. nonsteroidal anti-inflammatory drugs, NSAIDs) that is not compliant with the current guidelines [17,18,19,20]. According to several evidence-based guidelines, the most effective therapies are aerobic exercise, cognitive behavioral therapy (CBT), multimodal therapy [21], and amitriptyline [22]. Patients with FMS also frequently request complementary treatments [23,24]. The majority of patients with fibromyalgia syndrome, for example, use heat applications and rate them as one of the most effective treatment strategies [25]. Heat applications are also explicitly recommended as a self-management method in the current German medical S3 guidelines [26], but no specific recommendation is made for outpatient whole-body hyperthermia (WBH) due to the limited number of studies. WBH works by increasing the body core temperature to create an artificial fever-like state [27] and basically consists of three phases. In the so-called heating phase, the body core temperature is slowly raised until the target temperature is reached. After reaching the target temperature, the irradiation is stopped and the temperature is maintained for a defined period of time (plateau phase). This is followed by the resting phase, i.e., the patient is wrapped in blankets or foils to maintain the achieved core body temperature. Adverse effects were mostly physiological reactions to body heating and were only short-lived.

Previous studies revealed the first positive outcomes in terms of pain relief using mild water-filtered infrared-A (wIRA) WBH in FMS [28,29,30,31,32,33]. However, two of the cited studies were non-controlled [31,32], and three of the controlled trials were not randomized [29,30,33].

Based on these trials, our working group conducted a randomized sham-controlled trial in an outpatient setting, by creating an adequate sham condition with high credibility [34]. The key difference between the two conditions was that the subjects received significantly less heat by covering the device with an insulating foil. The outcomes of this present trial showed a significant reduction in pain intensity using mild wIRA whole-body hyperthermia after the intervention in comparison to the control group, indicating the potential of mild WBH as an effective therapy option for FMS patients. However, not all patients profited to the same or a comparable extent.

It is therefore of high relevance to gain further insight into the mechanisms of pain relief using mild WBH application by investigating possible predictors for the effectiveness of WBH. When looking at WBH studies, one sees, among other things, differences in the target temperature (38.1–38.5 °C) and the duration of the hyperthermia phases (e.g., plateau or resting phase) or sessions, which may be relevant for the outcome of the treatment [28,30,34]. Based on the study situation, the German guideline on WBH recommends a plateau phase of 15 min [35]. To sum up, the question arises: What influence does the intensity of pain have at the beginning of therapy and what is the effect of the duration of the plateau phase, i.e., the phase in which the body core temperature is kept at the target temperature within the 60 min treatment unit?

Furthermore, we aimed to use a qualitative research approach to develop an additional perspective on the intervention and its effects, as well as to gain new insights to best optimize WBH treatment in FMS. Patients’ perspectives enable us to provide an answer to the following main questions: How did patients experience the intervention? Did patients perceive changes as a result of the intervention?

## 2. Materials and Methods

*Design and procedure*. The monocentric, randomized, sham-controlled, single-blind, two-armed, parallel group trial designed as a mixed-methods approach was conducted from November 2020 to December 2021 at the Sozialstiftung Bamberg, Bamberg, Germany. The study was approved by the Ethics Committee of the Bayerische Landesärztekammer (BLÄK, approval number 20079), registered on ClinicalTrials.gov (https://clinicaltrials.gov/ct2/show/NCT05135936, accessed on 15 September 2023) and performed according to the Declaration of Helsinki by applying good clinical practice standards.

The main inclusion criteria were age between 18 and 70 years, a medically confirmed diagnosis of FMS according to the ACR 2016 criteria, and an average pain intensity of ≥ 4.0 measured using the Brief Pain Inventory (BPI). Participants who signed all the information consent forms and were medically assessed as eligible were randomized into two groups: the intervention and control group. The main exclusion criteria were severe somatic and psychiatric comorbidities and other chronic pain syndromes, as well as the intake of certain medications (opioids, cannabis, and immunosuppressive drugs). Participants with contraindications for hyperthermia such as increased body temperature (>37.5 °C) or heart failure, and previous experience with WBH were also excluded. More details can be found in Langhorst et al. 2023 [34].

*Participants*. Overall, 41 patients participated in the study by Langhorst et al. 2023 [31]. For the analyses of the present article, data from 20 participants of the intervention group (18 female) with ages ranging from 33.0 to 63.0 (M = 54.6; SD = 7.8) were subject to quantitative data analysis, and data from 10 participants of the intervention group (9 female) with ages ranging from 33.0 to 63.0 years (M = 51.8; SD = 9.7) were subject to a qualitative data analysis. Those 10 participants did not differ statistically from the other 20 participants with regard to all relevant variables (see Table 1).

*Intervention*. The participants of the intervention group received six sessions with mild wIRA-WBH within three weeks with at least one day between each session. One application lasted 60 min. For this purpose, the IRATHERM1000 (Von Ardenne Institut für Angewandte Medizinische Forschung GmbH, Dresden, Germany) device was used.

In accordance with the German guidelines for mild whole-body hyperthermia (WBH) [35], the desired body core temperature for each session in our current study was 38.5 °C, measured rectally using a device from Bluepoint Medical, which provided an accuracy of ±0.1 °C within the range of 25 °C to 50 °C. The irradiance level was initially set at 80% (1120 W/m^2^) during the heating-up phase. Once the target temperature was reached, the irradiance level was reduced to 40% (560 W/m^2^) to maintain the body core temperature until the end of the 60 min treatment session (plateau phase), if possible for a period of 15 min. This led to a further small increase in temperature in most of the subjects. After treatment, participants rested for about 30 min (resting phase). Subjects were permanently supervised by the performing therapist and a physician was always on call [34].

*Outcomes*. Besides demographic characteristics, which were captured only at baseline, clinical characteristics were recorded at baseline (week 0) and at follow-up measurements (week 12). Among other measurements that are described in Langhorst et al. 2023 [34], pain intensity as the primary outcome was measured using the Brief Pain Inventory (BPI), whose subscale describes a mean score ranging from 1 to 10 consisting of pain in the past 24 h (strongest–lowest–average) and current pain. Higher scores show higher average pain intensity. A pain reduction of 30% or more is considered to be clinically relevant [36]. At the intervention sessions (week 1–3), the durations of the heating-up and plateau phases were recorded.

Patients from the intervention group were asked to take part in a semi-structured telephone interview at week 12 (2 months after last intervention) because they could provide us with important insights into their experience with hyperthermia. The telephone was chosen for safety reasons for both participants and interviewers, as the COVID-19 pandemic had an effect at the time of data collection in 2021. There were 10 patients (n = 10) from intervention group that agreed to participate. No additional randomized or purposeful sampling procedure was undertaken. We did not exclude further participants. The breadth of the interview sample was sufficient for the epistemological interest of our mixed-methods design, which aimed to add subjective perspectives alongside quantitative data, as the interviews were information-rich cases and went into depth to provide the necessary information [37]. The participants varied in terms of relevant characteristics, e.g., age in years. Thus, different subjective perspectives and experiences could be examined.

For data collection, a semi-structured interview approach using an interview guideline (see Table S1) was conducted, on the one hand, to investigate specific topics in the interviews, as well as to consider prior knowledge [38]. On the other hand, this approach enabled new insights and let participants focus on their own topics and meanings [39,40]. The interview guideline was mainly developed deductively and included open-ended questions referring to research interests. The interviews were recorded following patient consent, transcribed verbatim, and anonymized [41]. The duration of each interview was approximately 30 min. The interviewers prepared interview protocols as field notes to document any peculiarities during the interviews.

**Figure 1:**
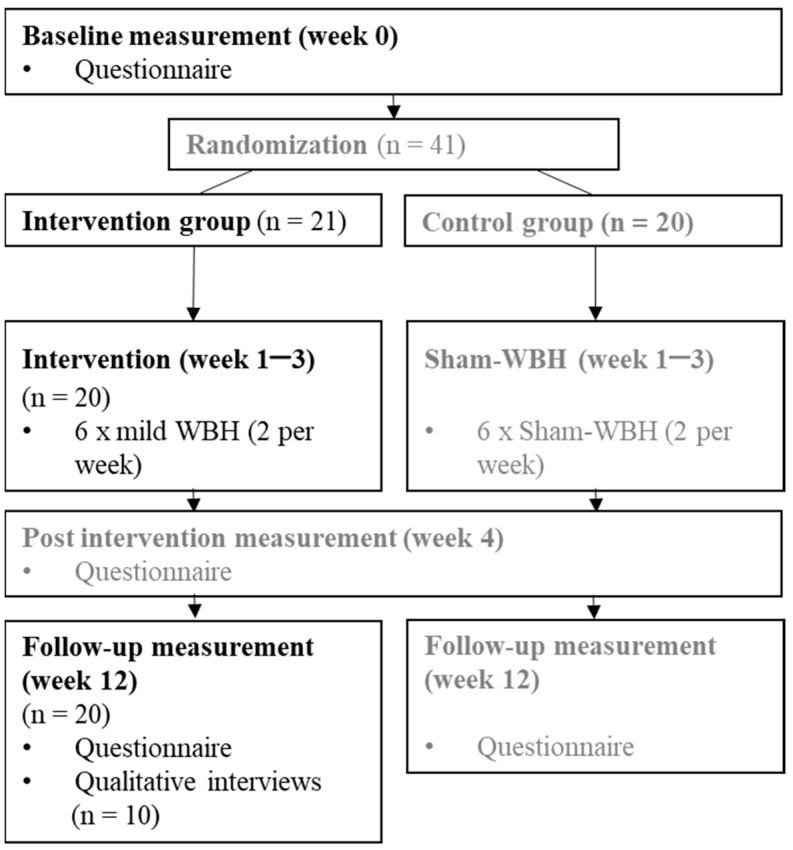
Flowchart. Note: n = number of cases; only data of the intervention group was analyzed, shown here in black font, referring to [34].

An overview of the study process with the respective measurement points can be found in Figure 1.

*Data analysis*. Intention-to-treat analyses were performed in Langhorst et al. 2023 [34]. For the present analysis, one participant was excluded due to missing values in the plateau phase.

*Quantitative analysis*. Multiple linear regression was used to analyze the quantitative data of the 20 participants from the intervention group. Multiple linear regression is a powerful tool for analyzing and predicting outcomes when you have multiple factors (independent variables) that may influence the dependent variable. It therefore extends the simple linear regression model, which involves just one independent variable. Its main goal is to find the best-fitting linear equation that represents the relationship between the dependent variable and the independent variables. Using various metrics (R-squared [R^2^], adjusted R^2^, *p*-values, F-statistic), the quality of the model can be assessed. These metrics help us to understand how well the independent variables explain the variance in the dependent variable. The analysis of the estimated coefficients reveals the relationship between the dependent variable and each independent variable (positive coefficients are equivalent to a positive relationship, and negative coefficients to a negative relationship) and the magnitude indicates the strength of the relationship [42].

*Qualitative interview analysis*. Reflexive thematic analysis was used to develop relevant themes and patterns in the subjective meanings of the participants. Interpretation and hermeneutic procedures enabled access to these meanings [43,44]. This approach is located in the field of symbolic interactionism since the knowledge of interest lied in the subjective views of the patients and data were obtained via communication [45]. The interdisciplinary research team from psychology, sociology, biology, and medicine used the software MAXQDA version 2022 for coding processes. Analytical categories (see Table S2) based on empirical data as well as on the specific research questions and prior considerations, a combination of inductive and deductive elements, i.e., data-driven and theory-driven coding, were chosen. During the process of analysis, the procedure and the results were discussed and reflected on in the research team to ensure validity, transparency, and intersubjectivity [46].

## 3. Results

### 3.1. Quantitative: Regression Analysis

Multiple regression analysis was used to test whether the mean duration of the plateau phase during hyperthermia treatment and baseline pain intensity (week 0) significantly predicted the pain intensity after intervention (week 12). A significant regression equation was found, F(2,17) = 9.83, *p* = 0.001, with an R2 of 0.546, i.e., the two predictors together explained 54.6% of the variance. Pain intensity at baseline significantly predicted pain intensity after intervention (β = 0.63, t(19) = 3.76; *p* = 0.002), as did tendentially the duration of the plateau phase (β = −0.30, t(19) = −1.78, *p* = 0.093), i.e., the longer the duration of the plateau phase, the lower the pain intensity afterward (and vice versa).

### 3.2. Qualitative Interviews

Patients with fibromyalgia in this study are affected by the physical, psychological, and social consequences of their disease in their daily lives, in particular by pain, sleep disturbances, chronic fatigue, etc. The narratives, e.g., P 01, P 03, P 10, indicate a burden and reduced quality of life: “I would be asleep if I didn’t have pain. The pain wakes me up” (P 10, female, 58 years)”; “On bad days I could do almost nothing, I was really tired and exhausted.” (P 03, female, 46 years); “I think there are two main things. Firstly, that I am constantly in pain and secondly that it is not being taken seriously by the environment, they say ‘Don’t be so dramatic’” (P 01, female, 61 years).

The patients report varying reasons for participating in the WBH study. The main reason for most of them was the hope for a long-term successful therapy of the pain symptoms. Previous therapies remain largely unsuccessful for most of the patients: “Because there was just a great hope that now maybe I would finally be helped, that one day the pain would go away” (P 08, female, 62 years).

The WBH application was experienced differently by the patients. The narratives vary from (partly, sometimes) negative experiences, i.e., unpleasant/bad feelings, stressful, painful, exhausting (P 04, P 07, P 08, P 10) to mixed (P 02, P 06) and positive experiences, i.e., (mostly, always) pleasant feelings (P 01, P 03, P 05, P 09).

“Very positive. Very positive. […] I really felt very well. Already since the first treatment. […] I did not have any circulatory problems, nor did my skin react in any way, nor anything else. Thus, I found this warmth extremely pleasant.”

(P 03, female, 46 years)

“Well, the first time I was there […] And it wasn’t so great. Because I felt that at some point at the end, so the last half hour I actually felt very unpleasant. So hardly bearable and I mean, I guess I just react to everything a lot.”

(P 07, female, 63 years)

The narratives suggest that even though some individuals here were classified as having mostly negative experiences with the applications, not all of their applications were in fact negative. Although the application as such is experienced differently across the interviews, a tendency toward generally positive experiences of the whole intervention can be drawn, as we can see in narratives about the wish for future hyperthermia applications as well: “So if it was offered to me, I would definitely do it again without any hesitation” (P 01, female, 61 years); “I would do it again like this at any time” (P 02, female, 50 years).

In two cases, there was no homogeneous picture in the narratives, i.e., a report of mixed experiences. In this regard, a change may occur during the treatment period, i.e., there may be differences between the different treatments, e.g., the first treatment being experienced as bad and later improvement during subsequent treatments, e.g., pleasant feelings (P 06). It was also reported by the patients that sometimes it seems that it depended on their own state, i.e., how they felt before the application and how their physical state of well-being was; if their well-being (physical and/or mental) was impaired, then also the application was not experienced well:

“Sometimes it was okay, it was more like a sauna. And two or three times I had to struggle a lot. I was also on the verge of breaking off. So, it was very different, depending on the day-to-day condition probably.”

(P 04, female, 33 years)

To better understand the general positive evaluation of the treatment, the perceived improvements due to hyperthermia are important to consider. The interviewees report about perceived improvements during and after the intervention. However, which effects and/or to what extent change was perceived differs. Nonetheless, the narratives in all interviews indicate that there is a perceived reduction in pain. In addition, better sleep behavior (therefore, being more rested during the day), generally better well-being, and in some cases, the possibility of more activity/movement in everyday life were reported:

“Yes, because the pain was also gone, a suffering was also gone […]. And yes, I felt freer and lighter and (…) more relaxed too. […] a bit more liberated and fuller of life and joy of living.”

(P 01, female, 61 years)

“Now it’s different […] it’s that things are easier for me now, much easier for me. Yes, I no longer have this daytime exhaustion, I also sleep really well. […] Yes, and the pain is there, but no longer at such a high level of pain and it no longer plays such a big role.”

(P 03, female, 46 years)

Among those who perceived positive effects, the time of occurrence varied and is reported differently, from or after the first intervention (the same day or the day after) to only after the last intervention, as we can see in the following quotations:

“Already during the first application I thought I was almost on cloud nine, because it went much, much better. […] So the pain was not gone, but it was considerably reduced.”

(P 01, female, 61 years)

“A few weeks after the study, I suddenly thought, huh? I don’t have any pain? I had then at least two weeks of no pain.”

(P 08, female, 62 years)

How long these positive effects lasted was very different. Regarding pain reduction, the duration varied from a short duration (a few hours after treatment up to one day; P 09, P 10), up to several days and weeks (P 01, P 04, P 05, P 06, P 07, P 08), or up to the time of interview (8 weeks after last intervention, P 02, P 03) was reported. Pain was back to pre-treatment levels in most patients, or still reduced in two cases:

“And two weeks after that, everything was back the same as before”;

(P 04, female, 33 years)

“So the change that is quite clear is actually this improvement in getting up in the morning and the sleep is more relaxing […] And actually it is still like that now.”.

(P 05, female, 54 years)

### 3.3. Mixed-Methods Perspective–Pain Reduction, Duration of Plateau Phase, and Improvement

By looking at the possible predictors and the patients’ narratives in the interviews, we were able to identify certain patterns. The quantitative results show that the longer the plateau phase lasts, the lower the pain intensity tends to be afterward (and vice versa). All 10 interviewees reported an improvement in pain at some point. Furthermore, the patients reported that the pain relief endured for an extended period, with two of them experiencing sustained relief until the 12-week follow-up interview. In addition, there was a clinically relevant pain reduction of 30% or more at week 12 in three of the patients interviewed. These cases were associated with a longer plateau phase lasting between 17.0 and 23.3 min (Table 1). The interviews also provide us hints about this possible connection from the patient’s point of view. P 04 describes her perception that the longer it took her to reach the plateau phase—which corresponds to a plateau phase that tends to be short—the worse she felt afterward, i.e., she experienced no improvement in pain, and in some cases, it even worsened: “I noticed that during the process, it also depended on how quickly I reached the goal, the temperature goal. So, the longer it took, the worse I felt” (P 04, female, 33 years).

These results thus indicate a tendency for the duration of the plateau phase to have a positive influence on the success of pain reduction. However, there are also deviations in individual cases in which no clinically relevant improvement in pain was achieved at week 12 despite a longer mean plateau phase.

## 4. Discussion

Firstly, our analysis revealed that two key factors together, namely, the duration of the plateau phase during hyperthermia treatment and the baseline pain intensity at week 0, did significantly predict the pain intensity following the intervention at week 12.

Secondly, patients had diverse experiences with hyperthermia treatment, with some finding it uncomfortable and distressing, while others found it enjoyable and helpful. Some individuals reported mixed feelings about the treatment. Overall, all patients reported improvements in pain reduction. Moreover, patients noted better sleep, enhanced overall well-being, and increased daily activity as a result of the treatment. The duration of these positive effects differed among patients, with some experiencing short-term relief and others enjoying longer-lasting benefits.

Thirdly, combining the qualitative and quantitative results, it appears that patients with a clinically relevant pain reduction of more than 30% had longer plateau phases (>15 min) and reported improvements that lasted longer.

Previous studies have also investigated the effect of mild wIRA-WBH on pain relief in FMS [28,29,30,31,32,33]. To the best of our knowledge, we are the first research group to examine predictors for pain relief using WBH in fibromyalgia and include patient perspectives by uisng qualitative interviews. In addition to the results of our main study [34] on pain reduction, we chose this mixed-methods approach for this underlying research interest.

The statistical analysis revealed the two predictors for pain reduction in our study: baseline pain intensity and duration of the plateau phase during WBH. It showed a positive relationship between pain intensity at baseline and at week 12 (i.e., the higher the pain intensity at baseline, the higher it was after treatment), but it is important to note that for most of the participants (14 out of 20) there was a notable decrease in pain intensity after treatment. The duration of the plateau phase was negatively correlated with pain intensity, arguing for a strong effect of a longer plateau phase on the reduction in pain, even 8 weeks after the treatment. Therefore, there should be further studies using longer plateau phases to investigate whether the recommended 15 min (current guideline) should be extended to produce even stronger pain relief in patients with FMS.

The qualitative results indicate that patients’ experiences of hyperthermia treatment varied. Some found it unpleasant, stressful, and/or painful, while others found it pleasant and beneficial. Some participants reported mixed experiences, which could change over the course of treatment and were influenced by their physical and mental states. However, the term “unpleasant experience” does not mean that the application did not bring any improvement. It means that the increase in temperature with its associated side effects was found to be unpleasant and distressing in most applications, but there may also have been applications that were experienced positively. In this regard, a crucial factor for the evaluation of the application could be the individual perception of the temperature increase or the time until the target temperature was reached. According to the patients, how one feels about the application can also depend on their personal day-to-day condition. In addition, the first application is sometimes experienced as the worst, as this is where the strain is perceived to be the highest. Another aspect for the evaluation of the application can be the perceived improvements. The timing of these perceived improvements varied from patient to patient and occurred in a range from immediately after the first treatment to only after the completion of the entire course of six treatments. The duration of these positive effects also varied, with some patients experiencing short-term relief, while others reported longer-lasting benefits. Importantly, however, the narratives show that all respondents had positive effects on their pain, but some of them concluded that this therapy was not worth the effort because the effects were not as long-term as hoped. For them, the cost–benefit relation is not balanced well enough. However, for all the variation in individual experiences with treatment, there was a general tendency toward positive evaluations, with many patients expressing a willingness to undergo hyperthermia treatment again.

Combining the qualitative and quantitative results, it seems that in our sample, patients’ clinically relevant reduction in pain of more than 30% was related to a longer plateau phases (>15 min), and those patients also reported longer-lasting improvements. The duration of the plateau phase is therefore quantitatively relevant. However, when looking at individual cases, there are exceptions. There are also patients who, on average, spent more than 15 min in the plateau phase but still did not experience significant improvement. This could be due to the fact that by week 12, there was no longer a clinically relevant pain reduction, since the effect had already receded, as shown in the interview study part. However, in these cases, the interviews indicate short-term effects that are no longer present to the same extent at week 12. In this regard, the main paper [34] also shows a clinically relevant pain reduction at week 4 in approximately 50% of the patients of the intervention group.

In this respect, this finding is consistent with an underlying study [34] that found by week 12, there was no significant reduction in pain intensity compared to the control group. However, pain intensity immediately after the intervention (week 4) was significantly reduced but apparently did not persist until the next measurement point for all patients. Nevertheless, there were patients who clearly demonstrated pain reduction even after 6 months [34] and benefited from the intervention in the long term. Here again, it becomes apparent that there are long-term improvements in individual patients.

All interviews indicate that there is a perceived reduction in pain. This improvement in pain perception is associated with diverse improvements in other areas. Passive body heating had a positive effect on the sleep patterns of women with fibromyalgia [47]. In the main study, it was observed that sleep quality, assessed using the Pittsburgh Sleep Quality Index (PSQI) questionnaire, tended to improve with the intervention [34]. Now, in the interviews, better sleep behavior and generally better well-being after the intervention were reported.

### Strengths and Limitations

The main strength of this study is the mixed-methods approach in the framework of a randomized controlled setting. By integrating the patient perspective, two viewpoints could be demonstrated in one approach. It must be emphasized that the number of cases overall in this study is small and only half of the sample was interviewed. However, looking at the general fibromyalgia population, our sample is quite representative regarding age and sex (mainly middle-aged women) [4]. Nevertheless, it can be assumed that the findings are applicable to our entire sample, as the group of interviewees did not differ from the overall group according to the parameters examined. However, these results may not be generalized to the FMS population in general. Also, future studies should examine how long-lasting these effects actually are and whether further WBH sessions are helpful for the maintenance of the improvements.

## 5. Conclusions

These findings indicate that despite varying individual experiences with the treatment, there was an overall tendency toward positive evaluations among patients. The duration of the plateau phase during hyperthermia treatment and the baseline pain intensity could be possible predictors of pain intensity after the intervention. The results of the study could hint that the plateau phase has an influence on long-lasting improvement (quantitative: reduction in pain score from the BPI; qualitative: reported duration of pain reduction). However, further investigation is necessary in this regard.

## Figures and Tables

**Table 1:** Patient results overview.

Patients	Interview (Yes = 1, No = 0) ^a^	Pain Intensity W0 ^a^	Pain Intensity W12 ^a^	Change in Pain Intensity [%] ^a,c^	Duration PlatP Mean All Sessions [Minutes] ^a^	Experience of the Intervention (Mainly) ^b^	Perception of Pain Reduction ^b^	Duration of Pain Reduction ^b^
1	1	5.3	5.3	0.0	15.0	Positive	Yes	Medium
2	1	4.6	2.8	−40.6	17.0	Mixed	Yes	Long
3	1	6.5	2.0	−69.2	23.3	Positive	Yes	Long
4	1	4.3	5.3	23.5	21.0	Negative	Yes	Medium
5	1	2.0	2.5	25.0	10.8	Positive	Yes	Medium
6	1	4.5	3.0	−33.3	18.5	Mixed	Yes	Medium
7	1	6.8	7.0	3.7	4.0	Negative	Yes	Medium
8	1	7.0	5.5	−21.4	9.8	Negative	Yes	Medium
9	1	7.0	6.4	−8.9	22.0	Positive	Yes	Short
10	1	4.8	4.3	−10.5	3.0	Negative	Yes	Short
11	0	5.0	3.6	−28.6	7.5	n.a.	n.a.	n.a.
12	0	5.8	4.0	−30.4	12.2	n.a.	n.a.	n.a.
13	0	4.3	1.8	−58.8	24.8	n.a.	n.a.	n.a.
14	0	5.8	3.0	−47.8	12.7	n.a.	n.a.	n.a.
15	0	4.8	2.3	−52.6	21.2	n.a.	n.a.	n.a.
16	0	4.8	4.5	−5.3	20.0	n.a.	n.a.	n.a.
17	0	5.3	5.3	0.0	13.5	n.a.	n.a.	n.a.
18	0	7.0	4.5	−35.7	19.7	n.a.	n.a.	n.a.
19	0	7.3	6.3	−13.8	5.0	n.a.	n.a.	n.a.
20	0	8.3	9.0	9.1	12.7	n.a.	n.a.	n.a.
Mean total	n.a.	5.5	4.4	−19.8	14.7	n.a.	n.a.	n.a.
Mean interview group	n.a.	5.3	4.4	−13.2	14.4	n.a.	n.a.	n.a.
Statistical difference (*t*-tests)	n.a.	*p* = 0.644	*p* = 0.989	*p* = 0.539	*p* = 0.930	n.a.	n.a.	n.a.

Note: grey background color indicates the interviewed patients; n.a. means not applicable; ^a^ quantitative results; ^b^ qualitative results; ^c^ change in pain intensity: positive values correspond to an increase, negative values to a decrease.

## Data Availability

Data will be provided by the corresponding author on request.

## Supplementary Materials

The following supporting information can be downloaded at: https://www.mdpi.com/article/10.3390/biomedicines11112949/s1, Table S1: Main topics, research aims, and exemplary questions from the interview guideline; Table S2: Main categories of the coding scheme.
