# Supplementary material for: Investigating the Influential Factors of Mild Water-Filtered Infrared-A Whole-Body Hyperthermia for Pain Relief in Fibromyalgia: A Mixed-Methods Approach Focusing on Predictors and Patient Perspectives
Source: Biomedicines. 2023 Nov 1;11(11):2949. doi: 10.3390/biomedicines11112949 (PMC10669402; doi:10.3390/biomedicines11112949)
Supplement: Supplementary file 1 [file biomedicines-11-02949-s001.zip › Table S1_Main topics and research aims_R.pdf]

Table S1. Main topics, research aims, and exemplary questions from the interview guideline

**Main topics and research aims**	**Exemplary questions and narrative stimuli**
➢ Experiences with the disease Fibromyalgia in everyday life	Please tell me about your personal experiences with Fibromyalgia in everyday life.<br>What have you done to treat your disease (the effects of the disease) so far?
➢ Reasons and motivation for participating in whole-body hyperthermia (WBH) therapy study and expectations	Why did you decide to participate in the study?<br>What were your expectations in advance?
➢ Experience with whole-body hyperthermia (WBH) therapy<br>• positive and negative aspects; satisfaction<br>• Suggestions for improvement	How did you like the therapy?<br>What did you find particularly good? What did you find less good?<br>What do you think should have been different and what else do you need for yourself?
➢ Perceived improvements due to therapy<br>• Changes in everyday life<br>• Perception of the effectiveness on or change of the disease/one's life	Can you please tell me if you have noticed any changes compared to before the therapy?<br>Did/does the therapy affect your life? The disease? If so, in what way? How does it manifest itself?
➢ Future use of whole-body hyperthermia (WBH) therapy	How likely do you think it is that you use again whole-body hyperthermia (WBH) therapy? Why?
