# Supplementary material for: Investigating the Influential Factors of Mild Water-Filtered Infrared-A Whole-Body Hyperthermia for Pain Relief in Fibromyalgia: A Mixed-Methods Approach Focusing on Predictors and Patient Perspectives
Source: Biomedicines. 2023 Nov 1;11(11):2949. doi: 10.3390/biomedicines11112949 (PMC10669402; doi:10.3390/biomedicines11112949)
Supplement: Supplementary file 1 [file biomedicines-11-02949-s001.zip › Table S2_Main categories of the coding scheme_R.pdf]

Table S2. Main categories of the coding scheme

Main codes	Sub codes
Fibromyalgia in daily life	Initial diagnosis
	Course of the disease
	Consequences of the disease / symptoms (physical, psychological, social level) in daily life
	Measures (therapies) taken against disease so far
Reasons for participation in the study	Motivation
	Expectations
Perception and experience of Hyperthermia therapy	Positive aspects and satisfaction
	Negative aspects and suggestions for improvement
Perceived changes	Improvements (psychological, physical, social)
	Worsening (psychological, physical, social)
	Time point of perceived change
	Duration of improvement / worsening
Future application of hyperthermia	Personal interest / aim of future application hyperthermia
	Cost-benefit consideration
	Compatibility of application with everyday life
